# Family-focused practice within a recovery framework: practitioners’ qualitative perspectives

**DOI:** 10.1186/s12913-017-2146-y

**Published:** 2017-03-24

**Authors:** B. Ward, A. Reupert, F. McCormick, S. Waller, S. Kidd

**Affiliations:** 1 School of Rural Health, Monash University, PO Box 666, Bendigo, VIC 3552 Australia; 2 Faculty of Education, Monash University, Clayton, VIC 3800 Australia; 3 Psychiatric Services, Bendigo Health, PO Box 126, Bendigo, VIC 3552 Australia; 4 School of Rural Health, Monash University, PO Box 397, Moe, VIC 3825 Australia

**Keywords:** Family-focused practice, Recovery, Health care

## Abstract

**Background:**

Family-focused practice (FFP) is an effective approach to supporting individuals with mental illness. ‘Recovery’ is also central to contemporary mental health care. However, there is a dearth of evidence about how the two concepts are related and subsequently implemented in practice. The aim of this study was to explore practitioners’ understandings and practices of FFP within a recovery framework.

**Methods:**

Purposive/snowball sampling was used to recruit and conduct qualitative interviews with 11 mental health practitioners in rural Australia. Concurrent sampling and data collection were informed by thematic analysis and continued until data saturation was reached.

**Results:**

Participants found it difficult to articulate their understandings of FFP within a recovery framework. Nonetheless they were able to describe practices that embodied family-focused recovery. Barriers to such practices included medical models of care, where there are often a shortage of skilled staff and high demands for care. Stigma (self and from others) and confidentiality were also identified as barriers to involving family members in recovery focused care.

**Conclusions:**

Family-focused recovery care is a priority in many high-income countries. A family-focused recovery framework is needed to assist service planners, practitioners, family members and those with mental health needs and ensure such care is embedded within practice guidelines.

**Electronic supplementary material:**

The online version of this article (doi:10.1186/s12913-017-2146-y) contains supplementary material, which is available to authorized users.

## Background

Internationally, mental health disorders represent a significant proportion of disability-adjusted life years (DALYs) [1]. Twenty percent of the population report having experienced mental illness in the preceding 12 months and 29% have experienced a mental disorder at some time during their lifetime [2]. Family support is critical to an individual’s recovery journey [3]. Family members are commonly involved in the care of and support for those with mental health concerns; thus mental illness has an effect on more than just the individual. Family-focused practice (FFP) is commonly used interchangeably with ‘family-oriented’, ‘family-sensitive’ and ‘family-centred’ but overall refers to an approach that acknowledges and addresses the needs of people with mental healthcare needs and their family [4]. A recent integrative review extended the field by identifying several core practices related to working with families including care planning, active emotional and social support and psychoeducation for family members within a coordinated care system [5].

Increasingly, governments across Europe, Australia and North America are advocating for a family centred model of practice when working with people with mental healthcare concerns. For instance, in Canada, The Rising to the Challenge government document advocates for the recognition and support of families in the recovery and well-being for those with a mental illness [6]. Additionally, there is a strong body of evidence to support FFP, as it has been shown to be effective for the individual with the illness as well as their family. Family-focused interventions have been shown to result in fewer relapses and a reduction in mood disorder symptoms for those with bipolar disorder [7] while acknowledging and supporting those with mental health care needs within the family context has been shown to result in a reduction in the family’s subjective burden of care and increases their level of self-care and emotional role functioning [4]. The efficacy and effectiveness of many family based approaches has been shown to improve prognosis and benefit carers by reducing stress associated with caregiving roles [8].

In recent years there has been a substantial investment in upskilling high-income countries’ mental health workforce in FFP. At the same time, FFP varies between professionals groups, countries and in different healthcare settings within the same country. Psychiatric nurses are less likely than social workers and psychologists to engage in FFP, a finding attributed to different philosophical paradigms [9]. Australian psychiatric nurses engage in higher FFPs, than Irish nurses, perhaps because standards and tools have been introduced in Australia to measure the effectiveness of family-focused mental health initiatives and interventions [10–12]. Others have argued that adult mental health services are less family-focused than other organisations based in the community, [9, 13], possibly due to the medical “patient” orientation of adult mental health services.

Simultaneously, the concept of recovery is another strong theme emerging in mental health services in many parts of the developed world. Anthony (1993, p. 13) describes recovery as

“… deeply personal, unique process of changing one’s attitudes, values, feelings, goals, skills and roles. It is a way of living a satisfying, hopeful and contributing life even with limitations caused by the illness. Recovery involves the development of new meaning and purpose in one’s life as one grows beyond the catastrophic effects of mental illness.”

Recovery typically focuses on the individuals’ journey. However, there are a range of other factors that influence recovery and these are mediated by connectedness [14] that typically includes family members. Hence, recovery journeys are relational and bidirectional as families influence an individual’s recovery journey and the recovery journey impacts the family. Despite this, families are typically represented in mental health practice “either as burdened carers, as causing the mental illness in a family member, as acting to sustain the mental illness or as contributing to relapse” [15]. Moreover, in terms of family relationships, those with mental healthcare needs are often represented as: (a) being passive recipients of family support, (b) actively caring for elderly parents and children and/or (c) participating in reciprocal, give and take family relationships [16]. Reupert et al. (2015) conclude that family interactions and roles offer the opportunity to both facilitate and impede recovery for those with a mental illness. Within a recovery paradigm, the family’s own recovery journey requires acknowledgment, in parallel with people with mental healthcare needs, which change over time [15]. For example, when a person is unwell, family relationships may be one sided and dependent while during the re-building phase, families need to “let go” and move from being a carer to a support person [16].

Internationally, the emphasis on FFP and likewise, recovery in mental health service policies is increasing [17]. While much conceptual work has been done on FFP and recovery separately, how the two concepts are intertwined and related is at present, unclear. While many mental health services subscribe to a philosophy of FFP, there is a dearth of evidence about how FFP is translated into practice, especially within a recovery framework. Defining and implementing FFP within a recovery framework is vague and not well understood. A soundly developed conceptual framework of recovery oriented FFP, grounded in the lived experiences of those with mental healthcare needs and families is needed, to establish practice standards and inform professional development.

## Aim

To address this gap, a study was undertaken to: (a) explore mental health practitioners’ understandings of recovery orientated FFP, (b) describe practitioners’ practices that embody family-focused, recovery orientated concepts and (c) identify the barriers and enablers to being recovery orientated when working within a family-focused approach.

## Methods

### Design and setting

A qualitative study, guided by a steering group of expert practitioners, was conducted to explore mental health practitioners’ understandings and practices around family-focused recovery orientated practice. The setting was a rural catchment area of 50,000 km^2^, servicing 230,000 people across the states of Victoria and NSW in Australia [18]. Across the region, levels of psychological distress, severe and profound disability are higher than that in comparable localities [18, 19].

### Procedure and sampling

The research questions and current literature [11, 20, 21] informed the development of the interview schedule. The semi-structured questions invited practitioners to describe their understanding of FFP within a recovery framework, and how these might be embodied in practice Additional file 1. The questions were pre-tested and modified following feedback from the steering group consisting of practitioners and managers. A purposive/snowballing approach was used to recruit “information-rich” [22] practitioners from a range of mental health service settings (e.g. acute inpatient, community, case-management) who work with people (and/or their families) with severe, persistent mental illness and complex needs. We sought practitioners with diverse backgrounds (e.g. social work, nursing, welfare workers) and levels of experiences.

### Participants

Eleven mental health practitioners were interviewed. Of these, eight provided services across at least half of the geographical region. As per Table 1, participants’ disciplinary background, workplace setting and years of experience working with families varied. For confidentiality reasons specific details about the role and location of each participant are not provided. There are no specific rules when determining an appropriate sample size in qualitative research. Instead data collection is generally continued until saturation has been reached [23]. Other comparable, interview based research with mental health practitioners in this field have involved six practitioners [24], fourteen nurses [25] and nine inpatient staff [26]; similar sizes to the eleven participants in this study.

**Table 1:** Participant demographics

Age	Median = 47 years (range: 23–59)
Gender	72% female, 28% male
Background	mental health nursing (5), social work (2), social/community welfare (2), psychology (1) occupational therapy (1)
Workplace setting	public acute care services = 4
	public community services = 5
	private practitioners = 2
Years working in mental health with families	Median = 6 years (range: 0.5–30)

### Data collection and analysis

The first author carried out all data collection and continued until thematic and information saturation had been reached. Interviews ranged from 40–90 min with an average of 57 min. Given the geographical distribution of participants, interviews were held either face-to-face or via the telephone. Interviews were audio-recorded and transcribed verbatim. All participants were provided a copy of their transcript for member checking; one added minor changes. The data were stored and managed in Nvivo [27]. Two researchers (BW and AR) independently read, re-read and coded the transcripts and field notes. Preliminary open coding was used to derive themes from the data and inform ongoing data collection [23]. Memos, field notes and visual representations of the data were used to facilitate team discussions and review of the emerging themes both within and across settings and participants’ disciplines. Differences were noted when identified [23, 28].

## Results

**Fig. 1:**
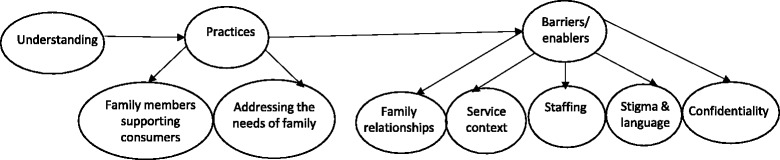
Practitioners’ understandings and practices: family-focused practice in a recovery framework

As per Fig. 1, key themes emerged in relation to understanding and synthesising FFP in a recovery framework, practices, barriers and enablers around the same. Each theme and sub-theme is described below and illustrated with participants’ quotes.

### Synthesizing family-focused practice and recovery

Overall, participants found it difficult to articulate what they understood as FFP within a recovery framework. Nonetheless there was an awareness about the importance of the family for their relative even though understandings of recovery were limited:

*We all know …that if you don’t engage the family meaningfully, peoples’ recoveries [are] really limited.* (nurse)

and

*We make sure that the person and the family is that which we hold sort of at the centre at all times, and that if we’re really looking at recovery we look at how we assist the family unit to work better together.* (social worker)

Providing a framework or definition that encapsulated the two concepts proved difficult and most tended to focus on what was meant by recovery alone. For instance, recovery was defined in terms of outcomes,

*Recovery in terms of becoming as well as you can … to, you know get your life sort of re-established or back on track.* (occupational therapist)

Conversely, while others saw recovery as a journey, they tended to refer to the journey as an individual journey of self-identity:

*Who is it that you want to be, who is it that you are now that this illness has come into your life in this way; how do you want to be in relation to this illness, it doesn’t have to define you.* (social worker)

Hope was also a key feature of practitioners’ definitions of recovery, though again family members and family relationships did not feature:

*If you don’t have hope and share hope with them that they can do it differently if they want to, then I think we have our obligations [as] therapists to assist people to try and hold onto hope somewhere.* (social worker)

There was some, albeit limited, acknowledgement of the family’s needs and their own recovery journey:

*So the family members having access to working with somebody as well, to look at recovery for them, for the ordeal that they’ve been through too.* (welfare worker)

### Enactments of family-focused practice within a recovery framework

Notwithstanding the difficulty in articulating a synthesized understanding of the recovery and FFP, participants were nonetheless able to detail specific practices that for them embodied family-focused recovery. These can be analysed into two sub-themes, (i) by encouraging and prompting the family to support their relative with the illness and (ii) by supporting the family and addressing their own needs.

i. Encouraging the family to support their relative
  Many participants saw family-focused recovery in terms of encouraging family members to support their relative in a very practical manner, for example:
*… whether it be administering the medications, or observing for side effects for them, or the direct care… yeah the more hands-on stuff, it’s taking the person to appointments, whether it be with us or a GP, collecting the medication from the pharmacy or bringing the medication in with the patient when they come in – yeah that’s probably what most of our role with the families would be, and yeah that’s probably it.* (nurse)
  This support also involved a focus on family dynamics:
*… if we’re really looking at recovery we look at how we assist the family unit to work better together.* (social worker)
  A focus on the family as a support system also had a reciprocal effect, by encouraging the person with mental healthcare needs to consider his or her family and their role in recovery, by asking for help or merely reflecting on how they might interact with them:
*…even if you don’t directly work with other family members in the room there has to be conversations about family and how the person who has the illness deals with family and how they deal with them.* (psychologist)
ii. Addressing the needs of family members
  In the first instance, addressing the needs of family members meant acknowledging the presence and role that the family played in a person’s recovery. After identifying these family relationships, participants described addressing the needs of families in several ways, including the provision of psychoeducation:
*I do quite a bit of … education with families when I have a client that has struggled to kind of express to family how life is for them. So sometimes I would invite family or carer in, often, parent or close relative to kind of explore a little bit with them, with the client in the room, about what it's like to live together.* (nurse)
  The importance of empowering people to lead discussions in their family about their mental health was a practice described by another participant as family orientated recovery.
*I’ve had a mum discuss with me several times about a conversation that she might have had or some things that she might have said to a child that is inappropriate for that child’s age. So obviously we would go into what things did you say, how could you have said that differently. (*community support worker)
  Another family orientated recovery strategy was promoting family connectedness and encouraging positive family interactions.
*Engaging together as a family; so when we look at her behavioural activation plan; we've included time, meaningful time with the children on that and so we’re looking at getting brokerage dollars to support them in buying bikes so they can go riding together.* (social worker)
  Supporting family members on an emotional level was also noted:
*… my role is, is about [saying] “yeah okay now when you're frustrated you know just remember that you know your mums got this illness and sometimes it's difficult for her to remember things and you know, and she becomes very tired and she needs the sleep. When you're feeling this way you know what tools can we, what can you learn to deal with that”.* (community support worker)

### Barriers and enablers to family-focused recovery practices

Participants identified a range of barriers and enablers to family-focused recovery practices including family relationships, service context, staffing, stigma and language and confidentiality. These were not always mutually exclusive.

#### Family relationships

While there was an acknowledgement that family support could positively influence a relative’s recovery, the family also had the potential to adversely impact on a person.

*… If part of their difficulties that they’re experiencing is related to the family, then it’s hard to work with the family… if there’s relationship problems with the family and it’s impacting on their current mood and mental state and other risks, then it’s going to be hard to approach the family and say this person is feeling this way because they’ve said that the relationship with you is not good.* (nurse)

#### Service context

The context of the service, especially the location of an organisation, had the potential to promote a family orientated recovery service:

*I've been in an ideal space where a medical practice is a family practice. So families wander into the room together anyway, because they're kind of used to it whereas in another service that I work it's always by invitation.* (nurse)

and

*I'm co-located there with community health services. People are much more comfortable to present to their session with their mum, with their dad, with their partner, with their kids, whereas I think here because it's seen as much more medical, and much more clinical it's fairly rare.* (social worker)

Conversely, other settings especially those located in acute hospitals, were not conducive to such work:

*The way we’re structured at the moment you have, you’ve got to do 2 hours doing rounds, so there’s 2 hours during the day where you go and do that, 2 hours where you sit out in the HDU* (High Needs Unit) *so that’s where you’re targeted to 4 patients…and when, they’ve got family members… [you] actually don’t get a lot of time…*

#### Staffing

Staffing shortages and a lack of trained practitioners in this area were other barriers. Similarly, another barrier for many was time to work with family in a recovery orientated manner.

*There's no possible way that we can do a proper assessment… talk to them about their illness, talk to them about their recovery plan, engage their family in a meaningful way. Our comprehensive assessment* [form] *is 14 pages long and out of all that there a box … a couple of inches for family.* (social worker)

#### Stigma and language

Self-stigma and shame dissuaded some people from involving their family in their recovery journey:

*There’s often real resistance on the part of consumers to having their families involved in a meaningful way because they have so much shame about their diagnoses and so much fear…* (welfare worker)

Likewise, the power of language and associated stigma in relation to family relationships was a considerable barrier for working with families in a recovery focused manner as typified in this particular case:

*He was a farmer. He was so insulted, humiliated, he was already really depressed and embarrassed about what had happened to the farm and they were on the brink of financial ruin then [to] have to have his wife called his carer… it was irretrievably broken down.* (welfare worker)

The stigma associated with being a ‘carer’ of someone with a mental health concern was raised again in this instance, referring to a public event:

*Very few of them [carers] turned up because … [they] didn’t want to be out in public and known to be as a carer of a mental illness person. And some of them don’t like their photos taken and put in the local paper.* (welfare worker)

Finally, confidentiality was problematic when working with those with mental healthcare needs and their families:

*I'm always reassuring people in a family that if I am speaking to another family member I’ll give them summary of exactly what I think I’ll share, so that they're – they don’t think I'm going to go out of kind of their boundary of what's okay.* (nurse)

## Discussion

This study provides new insights into mental health practitioners’ understanding of, and the practices that embody FFP within a recovery framework. Our results confirm that many mental health practitioners have a clear understanding of FFP and to a lesser degree, recovery [29]. However, most struggled to provide a merged or synthesized definition of these concepts. Despite this, several were able to describe their practices that demonstrated embodied family-focused recovery. Our findings suggest practitioners are aware of the intertwined relationship between people with mental healthcare needs and their family members and the subsequent impact on the mental wellbeing of all family members [30].

For participants here, family-focused recovery was operationalised in two ways, first by supporting the family to better assist their relative in their recovery, and secondly by addressing the needs of the family themselves. In the first instance, the pragmatic support that families provide and indeed, the reliance of practitioners for this support, especially around medication compliance, has been described elsewhere [31]. At the same time, practitioners in the current study noted the importance of promoting positive family dynamics when working with the people with mental health concerns as well as when working with family members. In comparison, Matos and Sousa (2004) found that FFP typically involved instrumental support in areas such as finances, food and medicine, rather than enhancing family relations [32]. Hence, this finding is an important advancement given the importance of the family environment in a person’s recovery.

One participant described how she would encourage and guide a person to talk to their family, including children, about their mental health concerns. While family psychoeducation is a long established practice, this is typically provided to the family by a practitioner or via some other medium (such as DVD or over the internet) [33]. Empowering a person to assume control over the psychoeducational process within their family is well aligned to a recovery approach. The process within families of making sense of a relative’s illness is important as it enables mutual support and constructive problem solving [34] and according to this participant was an important practice associated with a family-focused recovery approach.

Context appeared to be a factor in practitioners’ understandings and delivery of family-focused recovery practice though this appeared to be more related to the type of organisation (acute vs community) rather than being in a rural setting. Participants based in or associated with public acute settings were more focused on personal recovery and less likely to understand and describe relational recovery than those who had current/past experience in dedicated FFP roles. Practitioners in acute settings are often oriented to biomedical psychiatry where treatment focuses on symptoms rather than context of a person’s care [35]. In these settings the culture and language is often focused on mental health risk assessment and discharge because they are mandated and urgent tasks [36]. In addition, many people with mental healthcare needs and staff have experienced trauma [37, 38]. As an element of recovery oriented practice, trauma informed practice may be poorly understood; particularly in acute settings [37]. While key performance indicators can be problematic [39], they do not exist for family-focused recovery practice. Practitioners in acute settings may well be providing family-focused recovery care but it is likely to be embedded and integrated into medical tasks and incidental and so not documented or identified as a priority.

Leaders in acute settings with in-depth understanding of recovery oriented FFP might role model and advocate for family-focused recovery practice training to ensure the knowledge of the family is included in the formulation of care plans [40]. While many of the social barriers (e.g. family history) to family-focused recovery care are not easily modifiable, the health system factors such as lack of training and emphasis on family-focused recovery practice in acute service and discharge planning can be addressed. Reorienting these services to a non-medical model of recovery that is driven by people with mental healthcare needs is possible but needs health service support.

Many practitioners interviewed in this study were unable to articulate a grounded framework for family-focused recovery, which is perhaps indicative of the broader research in this area. Even though frameworks can assist practitioners to think critically and broadly about the interventions they provide and the evaluations they apply [41], it is not unusual for mental health practitioners to find it difficult to describe the theoretical (or otherwise) basis of their work [42]. Nonetheless, a family-focused recovery framework is important as it might be used to understand mental health problems, within the context of families. Such a framework could also be used to inform organisational and practitioners’ interactions with those with mental healthcare needs and their families, and their overall response to mental illness. Accordingly, it is imperative that future work is conducted to locate and position family-focused recovery in policy and practice guidelines.

Our study had limitations. It was confined to one rural geographical area, and participants in other settings may have different experiences. However, the locality is typical of many rural and remote areas where there are high levels of stigma (or added sensitivity to privacy) around mental illness, poor access to general and mental health services and higher levels of mental illness [43, 44]. The literature on FFP and recovery is growing rapidly [5, 29], but our understanding of, and research on, how to integrate these two complex philosophies in mental health service delivery is sparse. Further research is needed to theorize FFP within recovery frameworks and explore how these concepts can be embedded in a range of practice settings.

While the current study confirmed prior notions of family-focused recovery practice for addressing the emotional and pragmatic needs of the families [45], our research has provided new insights into practitioners’ understanding of family-focused recovery. Important findings that advance the field is that practices related to family-focused recovery involves promoting positive family dynamics and empowering the person so they could hold discussions within their family about their mental health concerns. Such findings have training implications for example, coaching practitioners to support people to guide conversations about their mental health in their families. It is important to note however, that the practitioners here did not consider family members’ own recovery journeys, in regard to maintaining hope, reconnecting and overcoming secondary trauma (as theorised previously) [15]. These specific gaps suggest a training need around how practitioners might support a family’s own recovery journey.

## Conclusions

Family-focused recovery practice is dynamic [5]. It needs to respond to the changing nature of the recovery journey for people with mental healthcare needs and their family members. This is an ongoing challenge for health services and the effectiveness of any family-focused recovery interventions should be monitored using validated tools that enable us to measure change. The implementation of national reforms in the Australian mental health care system [46] need to prioritise family-focused recovery practice within and across acute and community health care services.

## Additional file

Additional file 1: Interview schedule – mental health practitioners. (DOCX 33 kb)

## Abbreviation

FFP: Family-focused practice

## References

[1] Whiteford HA, Degenhardt L, Rehm J, Baxter AJ, Ferrari AJ, Erskine HE, Charlson FJ, Norman RE, Flaxman AD, Johns N (2010). Global burden of disease attributable to mental and substance use disorders: findings from the Global Burden of Disease Study. Lancet.

[2] Steel Z, Marnane C, Iranpour C, Chey T, Jackson JW, Patel V, Silove D (2014). The global prevalence of common mental disorders: a systematic review and meta-analysis 1980–2013. Int J Epidemiol.

[3] Onwumere J, Bebbington P, Kuipers E (2011). Family interventions in early psychosis: specificity and effectiveness. Epidemiol. Psychiatr. Sci..

[4] Foster K, O’Brien L, Korhonen T (2012). Developing resilient children and families when parents have mental illness: a family-focused approach. Int J Ment Health Nurs.

[5] Foster K, Maybery D, Reupert A, Gladstone B, Grant A, Ruud T, Falkov A, Kowalenko N (2016). Family-focused practice in mental health care: An integrative review. Child Youth Serv.

[6] Government of Manitoba: Rising to the challenge: A strategic plan for the mental health and well-being of Manitobans. 2011. [www.gov.mb.ca/healthyliving/mh/docs/challenge.pdf]. Accessed 18 Jan 2017.

[7] Glynn SM, Cohen AN, Dixon LB, Niv N (2006). The potential impact of the recovery movement on family interventions for schizophrenia: opportunities and obstacles. Schizophr Bull.

[8] Falloon IR (2003). Family interventions for mental disorders: efficacy and effectiveness. World Psychiatry.

[9] Maybery D, Goodyear M, O’Hanlon B, Cuff R, Reupert A (2014). Profession differences in family-focused practice in the adult mental health system. Fam Process.

[10] Maybery D, Goodyear M, Reupert A (2012). The family-focused mental health practice questionnaire. Arch Psychiatr Nurs.

[11] Goodyear M, Hill T-L, Allchin B, McCormick F, Hine R, Cuff R, O’Hanlon B (2015). Standards of practice for the adult mental health workforce: Meeting the needs of families where a parent has a mental illness. Int J Ment Health Nurs.

[12] Grant A, Goodyear M, Maybery D, Reupert A: Differences Between Irish and Australian Psychiatric Nurses’ Family-Focused Practice in Adult Mental Health Services. Arch. Psychiatr. Nurs., 30(2):132–137. 10.1016/j.apnu.2015.07.005 10.1016/j.apnu.2015.07.005 26992860

[13] Lauritzen C, Reedtz C, Van Doesum KT, Martinussen M (2014). Implementing new routines in adult mental health care to identify and support children of mentally ill parents. BMC Health Serv Res.

[14] Collins J, Ward BM, Snow P, Kippen S, Judd F: Compositional, Contextual, and Collective Community Factors in Mental Health and Well-Being in Australian Rural Communities. Qualitative Health Research 2016. doi:10.1177/1049732315625195 10.1177/1049732315625195 PMC5347352 26848083

[15] Wyder M, Bland R (2014). The recovery framework as a Way of understanding Families’ responses to mental illness: balancing different needs and recovery journeys. Aust Soc Work.

[16] Reupert A, Morgan DMB, Reupert A, Maybery D, Nicholson J, Gȍpfert M, Seeman M (2015). E-learning professional development resources for families where a parent has a mental illness. Parental psychiatric disorder: Distressed parents and their families.

[17] Nicholson J, Reupert A, Grant A, Lees R, Maybery D, Murdoch E, SkogØy B, Stavnes K, Diggins M, Reupert A, Maybery D, Nicholson J, Gȍpfert M, Seeman M (2015). The policy context and change for families living with parrental mental illness. Parental psychiatric disorder: Distressed parents and their families.

[18] Medicare Local catchment - Loddon Mallee-Murray [http://www.myhealthycommunities.gov.au/medicare-local/ml214] Accessed 3 Nov 2016.

[19] Department of Health: 2012 Regional health status profiles - Loddon Mallee region. In. Melbourne: Modelling, GIS and Planning Products Unit, Victorian Government Department of Health; July 2013. [https://www2.health.vic.gov.au/getfile/?sc_itemid=%7b8236947F-C27B-4C75-9A74-A6BB8BE17B74%7d&title=Loddon%20Mallee%20Region]. Accessed 3 Nov 2016.

[20] Reupert A, Maybery D, Nicholson J, Gȍpfert M, Seeman M (2015). Parental psychiatric disorder: Distressed parents and their families.

[21] Maybery D, Reupert A, Kent P, Goodyear M, Crase L (2009). Prevalence of parental mental illness in Australian families. Psychiatr Bull.

[22] Patton MQ (1990). Qualitative evaluation and research methods 2nd ed..

[23] Liamputtong P, Ezzy D (2005). Qualitative Research Methods.

[24] Reupert A, Maybery D (2011). Programmes for parents with a mental illness. J Psychiatr Ment Health Nurs.

[25] Grant A, Reupert A (2016). The impact of organizational factors and government policy on psychiatric Nurses’ family-focused practice with parents Who have mental illness, their dependent children, and families in Ireland. J Fam Nurs.

[26] O’Brien L, Brady P, Anand M, Gillies D (2011). Children of parents with a mental illness visiting psychiatric facilities: perceptions of staff. Int J Ment Health Nurs.

[27] QSR International: NVivo qualitative data analysis software. In., Version 10, 2012 edn: QSR International Pty Ltd.; 2012.

[28] Strauss A, Corbin J. Basics of Qualitative Research: Techniques and Procedures for Developing Grounded Theory. Thousand Oaks: Sage Publications; 1998.

[29] Slade M, Amering M, Farkas M, Hamilton B, O’Hagan M, Panther G, Perkins R, Shepherd G, Tse S, Whitley R (2014). Uses and abuses of recovery: implementing recovery-oriented practices in mental health systems. World Psychiatry.

[30] Reupert A, Maybery D (2014). Practitioners’ experiences of working with families with complex needs. J Psychiatr Ment Health Nurs.

[31] Marsh D (1998). Serious mental illness and the family.

[32] Matos AR, Sousa LM (2004). How multiproblem families try to find support in social services. J Soc Work Pract.

[33] Bäuml J, Froböse T, Kraemer S, Rentrop M, Pitschel-Walz G (2006). Psychoeducation: a basic psychotherapeutic intervention for patients with schizophrenia and their families. Schizophr Bull.

[34] Solantaus T, Reupert A, Maybery D, Reupert A, Maybery D, Nicholson J, Gȍpfert M, Seeman M (2015). Working with parents who have a psychiatric disorder. Parental psychiatric disorder: Distressed parents and their families.

[35] Kidd S, Kenny A, McKinstry C (2015). The meaning of recovery in a regional mental health service: an action research study. J Adv Nurs.

[36] Anthony W, Huckshorn K. Principled Leadership in Mental Health Systems and Programs, vol..Boston Boston University Center for Psychiatric Rehabilitation; 2008.

[37] Department of Health: Recovery-oriented practice: Literature review. In. Melbourne Victorian Government 2011.

[38] Lee J, Daffern M, Ogloff JRP, Martin T (2015). Towards a model for understanding the development of post-traumatic stress and general distress in mental health nurses. Int J Ment Health Nurs.

[39] Henderson C, Klimas J, Dunne C, Leddin D, Meagher D, O’Toole T, Cullen W (2014). Key performance indicators for mental health and substance use disorders: a literature review and discussion paper. Ment Health Subst Use.

[40] Perkins R, Slade M (2012). Recovery in England: transforming statutory services?. International review of psychiatry.

[41] James EL, Fraser C, Talbot L (2007). Vulnerable children in families affected by parental mental illness: The role of theory in programme development. Vulnerable Children Youth Stud.

[42] Reupert A, Goodyear M, Eddy K, Alliston C, Mason P, Maybery D, Fudge E: Australian programs and workforce initiatives for children and their families where a parent has a mental illness. Australian e-Journal for the Advancement of Mental Health 2009, 8(3):277–285. 10.5172/jamh.8.3.277

[43] Clement S, Schauman O, Graham T, Maggioni F, Evans-Lacko S, Bezborodovs N, Morgan C, Rüsch N, Brown JSL, Thornicroft G (2015). What is the impact of mental health-related stigma on help-seeking? A systematic review of quantitative and qualitative studies. Psychol Med.

[44] Ward B, Humphreys J, McGrail M, Wakerman J, Chisholm M (2015). Which dimensions of access are most important when rural residents decide to visit a general practitioner for non-emergency care?. Aust Health Rev.

[45] Reupert A, Maybery D, Cox M, Scott Stokes E (2015). Place of family in recovery models for those with a mental illness. Int J Ment Health Nurs.

[46] Hickie I (2015). Time to implement national mental health reform. Med J Aust.

