# Supplementary material for: Family-focused practice within a recovery framework: practitioners’ qualitative perspectives
Source: BMC Health Serv Res. 2017 Mar 24;17:234. doi: 10.1186/s12913-017-2146-y (PMC5364722; doi:10.1186/s12913-017-2146-y)
Supplement: Additional file 1: — Interview schedule – mental health practitioners. (DOCX 33 kb) [file 12913_2017_2146_MOESM1_ESM.docx]

**Interview schedule – mental health workers**

**Demographics**

**Gender:**

Male  Female **Age**:______(in years)

**Which of the following best describes the location of your workplace?**

Regional City  Rural  Remote

**What is your professional discipline**?

Psychologist  Social worker

Mental health nurse  Welfare worker

Other: please specify: _______________________

**What area of practice do you currently work in?**

Location _____________________Public or NGO or Private

**Employment status**

Full time  Part time  Other please specify:

**Year of experience working in mental health**

Between 0-5 years  Between 0-10 years  Over ten years of experience

**Year of experience working with families of patients/clients/consumers**

Between 0-5 years  Between 0-10 years  Over ten years of experience

What do you think is the impact of a consumer’s mental illness on family members?

What (if any) do you think your role is in working with families of patients with mental illness?

How (if at all) do work with family members of those with a mental illness?

What is your understanding of recovery?

How might family focused practice and recovery models be integrated?

What assists you in working with the family?

What (if any) guidelines/resources/training inform your work with family members?

What (if anything) stops you from supporting or working with a consumer’s family?

If we interviewed children, what do you think the children would say in regard to your family focused practice?
